# 909 Socioeconomic Disparities of the Inhalation Injury Patient Population

**DOI:** 10.1093/jbcr/iraf019.440

**Published:** 2025-04-01

**Authors:** Christopher Fedor, Hilary Liu, José Arellano, Mare Kaulakis, Garth Elias, Alain Corcos, Matthew Siedsma, Jenny Ziembicki, Francesco Egro

**Affiliations:** University of Pittsburgh School of Medicine; University of Pittsburgh Medical Center; University of Pittsburgh Medical Center; University of Pittsburgh School of Medicine; University of Pittsburgh Medical Center; University of Pittsburgh Medical Center; University of Pittsburgh Medical Center; University of Pittsburgh Medical Center; University of Pittsburgh Medical Center

## Abstract

**Introduction:**

Socioeconomic status has been consistently linked to both the incidence of house fires and the severity of burns and inhalation injuries resulting from these fires. Neighborhoods with lower income levels experience significantly higher rates of residential fires likely due to factors such as overcrowding, poor housing conditions, and lack of fire safety devices. Because inhalation injuries can be life-threatening, any social or structural barriers can lead to exacerbation of socioeconomic or racial disparities. This study aims to capture trends in the management, outcomes, and potential disparities that exist for patients suffering inhalation injuries.

**Methods:**

Inhalation injuries diagnosed on fiberoptic bronchoscopy were retrospectively reviewed using patient records from structure fires managed at a single tertiary care ABA-certified burn center (January 2012 - January 2024). Patient zip codes were matched with US Census data to estimate the level of urbanicity (Rural-urban community area codes, RUCA) as well as estimated level of social vulnerability during emergency situations (social vulnerability index, SVI). Patients were categorized into one of three groups: low (< 0.5), high (≥ 0.5 and < 0.75), or very high (≥ 0.75) SVI. Multivariate regression analyses were employed to adjust for age and percent total body surface area of cutaneous burns.

**Results:**

184 cases of confirmed inhalation injury were included in the study. 91.7% of patients were White and 8.3% identified as Black. 79.9% of patients were from urban communities (RUCA < 4). 19.6% were of low SVI status, 27.7% of high, and 52.7% of very high social vulnerability. Black patients were more likely to live in high SVI neighborhoods (p=0.006). From regression analyses, there were no observed differences in the ventilator days, hospital length of stay, nor the rate of excision and graft procedures. TBSA remained the only significant predictor of increased ventilator and hospital days (p< 0.001). Complication and mortality rates also neglected to reach statistical significance when compared across SVI groups (all p>0.05).

**Conclusions:**

Most patients who sustain inhalation injuries come from communities at risk of facing additional challenges during times of emergency. This is reflected by a high social vulnerability index score. Yet, despite this, higher SVI did not seem to impact clinical outcomes suggesting that emergency services and burn care teams can deliver equitable treatment, provided that resources were available. Addressing disparities in this field will include preventive measures in high-risk areas.

**Applicability of Research to Practice:**

While there were no differences among patients of different SVI levels, the fact remains that the majority of patients in the cohort were from at-risk communities. Thus, awareness and advocacy must be maintained to reduce fire risks.

**Funding for the Study:**

N/A

